# Post‐sensitization administration of non‐digestible oligosaccharides and *Bifidobacterium breve* M‐16V reduces allergic symptoms in mice

**DOI:** 10.1002/iid3.101

**Published:** 2016-03-24

**Authors:** Betty C. A. M. van Esch, Suzanne Abbring, Mara A. P. Diks, Gemma M. Dingjan, Lucien F. Harthoorn, A. Paul Vos, Johan Garssen

**Affiliations:** ^1^ Faculty of ScienceDivison of PharmacologyUtrecht Institute for Pharmaceutical SciencesUtrecht UniversityUtrechtThe Netherlands; ^2^ Nutricia ResearchUtrechtThe Netherlands; ^3^ Nutricia ResearchNutricia Advanced Medical NutritionUtrechtthe Netherlands

**Keywords:** acute allergic skin response, *Bifidobacterium breve* M‐16V, cow's milk allergy, FOS, fructo‐oligosaccharide, galectin‐9, immunoglobulins, inulin, mast cell, non‐digestible oligosaccharides, prebiotics, probiotics, regulatory T‐cells, synbiotics, T cell subsets, Th1‐cells, short chain fatty acids, propionic acid

## Abstract

To support dietary management of severe cow's milk allergic infants, a synbiotic mixture of non‐digestible oligosaccharides and *Bifidobacterium breve* M‐16V (*B. breve*) was designed from source materials that are completely cow's milk‐free. It was investigated whether this specific synbiotic concept can reduce an established food allergic response in a research model for hen's egg allergy. Mice were orally sensitized once a week for 5 weeks to ovalbumin (OVA) using cholera toxin (CT) as an adjuvant. Non‐sensitized mice received CT in PBS only. Sensitized mice were fed a control diet or a diet enriched with short‐chain‐ (scFOS) and long‐chain fructo‐oligosaccharides (lcFOS), *B. breve* or scFOSlcFOS + *B. breve* for 3 weeks starting after the last sensitization. Non‐sensitized mice received the control diet. Anaphylactic shock symptoms, acute allergic skin responses and serum specific IgE, mMCP‐1 and galectin‐9 were measured upon OVA challenge. Activated Th2‐, Th1‐cells and regulatory T‐cells were quantified in spleen and mesenteric lymph nodes (MLN) and cytokine profiles were analyzed. Short chain fatty acids (SCFA) were measured in ceacal samples. The acute allergic skin response was reduced in mice fed the scFOSlcFOS + *B. breve* diet compared to mice fed any of the other diets. A reduction in mast cell degranulation (mMCP‐1) and anaphylactic shock symptoms was also observed in these mice. Unstimulated splenocyte cultures produced increased levels of IL10 and IFNg in mice fed the scFOSlcFOS + *B. breve* diet. Correspondingly, increased percentages of activated Th1 cells were observed in the spleen. Allergen‐specific re‐stimulation of splenocytes showed a decrease in IL5 production. In summary; post‐sensitization administration of scFOSlcFOS + *B. breve* was effective in reducing allergic symptoms after allergen challenge. These effects coincided with changes in regulatory and effector T‐cell subsets and increases in the SCFA propionic acid. These results suggest immune modulatory benefits of dietary intervention with a unique combination of scFOSlcFOS + *B. breve* in established food allergy. Whether these effects translate to human applications is subject for ongoing clinical studies.

## Introduction

Food allergy is a major health problem for children living in western countries although it is not restricted to developed countries. Cow's milk allergy can be managed by avoidance of the culprit food or the use of alternative formulas based on hypo‐allergenic proteins, protein fragments or amino acids. An emerging treatment for patients with persistent IgE‐mediated food allergy is the use of allergen specific immunotherapy. Several routes of administration are being investigated, including oral, sublingual, epicutaneous, and subcutaneous. Food allergen specific immunotherapy aims to induce oral tolerance to the offending food, redirecting the allergic immune response from a Th2 type response to a Th1 or regulatory T‐cell response. Oral immunotherapy has been demonstrated to desensitize the majority of food allergic patients in observational studies and randomized controlled trials 1, 2, 3, 4. However, main concerns with allergen specific immune modulation are the high incidence of adverse allergic reactions during treatment, especially in severe cow's milk allergic patients 5, 6.

The use of amino acid based formulas in severe cow's milk allergy has been proven to provide safe and rapid symptom relief 7. An approach to stimulate oral tolerance acquisition and/or outgrowth of the allergic disease could be a useful extension of the functionality of amino acid formulas, but given the sensitivity of the patient group, the challenge is to achieve this safely without inducing adverse reactions.

The developing gut microbiome in early life has been shown to be different in cow's milk allergic children compared to healthy controls 8, 9, 10 and this microbial population is suggested to play a role in cow's milk allergy and long‐term development of allergies 11. Studies have demonstrated possible benefits of modulating the gut microbiome on atopic dermatitis using prebiotics and/or probiotics 12, 13, 14, 15, 16. In mice, a specific strain of mice which are predisposed to food allergy showed a different gut microbiota compared to wild‐type mice and adoptive transfer of the microbiota of the food allergy susceptive mice induced food allergic symptoms in recipient mice 17. Based on these data and the wide body of literature on immunomodulatory effects of commensal and/or probiotic bacteria, it can be hypothesized that interventions targeting the intestinal microbiome could modulate allergic immune responses away from a T‐helper 2 response by modulating the immunological “milieu,” even in the absence of any (modified) allergens. According to this hypothesis, an amino acid based formula with a mixture of non‐digestible oligosaccharides and probiotics (synbiotics) could have beneficial effects in severe allergic infants. In support of this concept, previous studies showed reduced allergic symptoms in mice fed a synbiotic mixture containing lactose‐derived non‐digestible oligosaccharides and *Bifidobacterium breve* M16‐V (*B. breve*) during oral sensitization and challenge, starting 14 days before the first sensitization 18, 19, 20. The effectiveness of this synbiotic was shown in a preventive setting; however, this mixture is not suitable for use in severe allergic children due to the presence of milk‐derived ingredients.

A recently developed amino acid based formula with a specific synbiotic concept was proven to be safe and well tolerated and to promote normal growth in healthy term infants 21 and cow's milk allergic infants 22. These synbiotics, containing short‐chain‐ and long‐chain fructo‐oligosaccharides, (scFOSlcFOS) and *B. breve*, were carefully selected to be free of cow's milk and cow's milk‐derived source materials to ensure the safety of the amino acid based formula for the intended population.

In the current study, it was investigated whether diets supplemented with plant‐derived scFOS (oligofructose) and lcFOS (long‐chain inulin) and/or *B. breve* have potential to reduce allergic responses in mice. To investigate the capacity of the diets to redirect an established allergic response, similar to the situation in established severe cow's milk allergy, the supplemented diets were fed to already sensitized mice. A combination of *in vivo* and *in vitro* functional and mechanism‐related outcome parameters was analyzed to quantify the immunomodulatory effects.

## Material and Methods

### Chemicals

Ovalbumin (Grade V) was obtained from Sigma (St. Louis, MO). Cholera toxin was purchased from Quadratech Diagnostics (Epsom, UK). Phosphate buffered saline (PBS) was obtained from BioWhittaker (Verviers, Belgium). Anti‐IgE was obtained from Pharmingen (Alphen a/d Rijn, the Netherlands). All antibodies used for flow cytometric analysis, galectin‐9, and cytokine analysis were obtained from BD biosciences (Breda, the Netherlands), with the exception of Forkhead box P3 (Foxp3)‐APC which was obtained from eBioscience (San Diego, CA).

### Diet

Semi‐purified cow's milk protein‐free AIN‐93G‐based diets were composed and mixed with non‐digestible oligosaccharides and/or *B. breve* by Ssniff Specialdiaten (Soest, Germany). The probiotic strain, *B. breve* (freeze dried on maltodextrin carrier and conserved at −20°C), was obtained from Morinaga (Morinaga Milk Industry, Tokyo, Japan) and 2 × 10^9^ colony‐forming units (CFU)/g was mixed through the diets and pressed into pellets. The prebiotic diet was designed for the use in an amino‐acid based formula, which is prescribed for the dietary management of moderate to severe cow's milk allergic infant and children who do not tolerate hydrolyzed infant formula. Therefore, the prebiotic diet was derived from cow's milk free source material. In this study, the non‐digestible oligosaccharides consisted of 1% (w/w) of a 9:1 (w/w) mixture of short‐chain fructo‐oligosaccharides (scFOS: oligofructose; Raftilose P95, Orafti, Wijchen, the Netherlands; >95% degree of polymerization [DP] < 6) and long‐chain fructo‐oligosaccharides (lcFOS: long chain inulin; Raftiline HP, Orafti, Wijchen, the Netherlands; average DP 23 or higher, <1% DP < 5) derived from chicory inulin (Raftiline HP, Orafti, Wijchen, the Netherlands). In total, four diets were prepared: control, scFOSlcFOS, *B. breve*, and synbiotic‐supplemented diet (scFOSlcFOS + *B. breve*) with comparable carbohydrate composition in the diets. The diets were stored at −20°C prior to use.

### Animals

Animal use was performed in accordance with guidelines of the Animal Ethics Committee of DEC consult. Four‐week‐old‐specific pathogen‐free female C3H/HeOuJ mice (Maastricht, the Netherlands; *n* = 8/group) were fed a cow's milk protein‐free AIN‐93G diet or a diet supplemented with *B. breve*, scFOSlcFOS, or the synbiotic concept containing scFOSlcFOS + *B. breve*.

### Experimental set‐up: dietary interventions, oral sensitization, and challenge of mice

**Figure 1:**
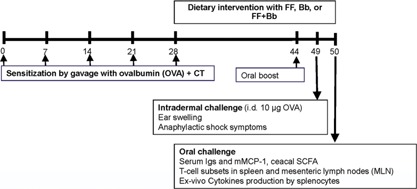
Schematic overview of the experimental set‐up; feeding the dietary intervention scFOSlcFOS (FF), *B. breve* (Bb) or the combination scFOSlcFOS + *B. breve* (FF + Bb) to already sensitized mice (Treatment setting) and parameters that were analyzed. OVA, ovalbumin; CT, cholera toxin; mMCP‐1, mouse mast cell protease‐1; scFOSlcFOS, short‐chain fructo‐ and long‐chain fructo‐oligosaccharides (9:1).

An overview of the experimental set‐up is provided in Figure 1. Mice were randomized over five groups, fed the control diet and sensitized intragastrically (i.g.) on day 0, 7, 14, 21, and 28 using a blunt needle with 20 mg ovalbumin (OVA) in 0.5 mL PBS using 10 µg cholera toxin (CT; Quadratech Diagnostics, Epsom, UK) as an adjuvant. Non‐sensitized mice received cholera toxin only (10 µg/0.5 mL PBS). On day 28, regular diets were changed for to the intervention diets. After the last sensitization, on day 28, mice were fed the control, scFOSlcFOS, *B. breve* or the synbiotic diet supplemented with scFOSlcFOS + *B. breve* for 3 weeks until sacrifice. Non‐sensitized mice received the control diet only. On day 44, an oral boost (20 mg OVA + CT) was given to be able to quantify OVA‐specific immunoglobulin responses. Five days later, all mice were challenged intradermally (i.d.) in the ear pinnae of both ears with 10 µg OVA in 20 µL PBS to determine the acute allergic skin response and anaphylactic shock symptoms. One day later, the mice were challenged i.g. with 50 mg OVA in 0.5 mL PBS and 18 h after the oral challenge blood samples were collected and centrifuged at 14,000*g* for 15 min. Sera were stored at −70°C until further analysis. Mice were sacrificed and samples were collected for ex vivo analysis.

In relation to a previous study with synbiotic diet containing milk derived non‐digestible oligosaccharides 18 a dietary intervention scFOSlcFOS + *B. breve* was investigated in an allergy prevention setting. Mice were fed the synbiotics during sensitization and challenge (from day 14 until the end of the experiment). The acute allergic skin response, anaphylactic shock symptoms, allergen‐specific IgE, and galectin 9 were measured in this preventive set‐up (Supplement Fig. S1).

### Acute allergic skin response and anaphylactic shock score

The acute allergic skin response was measured in duplicate for both ears 1 h after i.d. allergen challenge using a digital micrometer (Mitutoyo, Veenendaal, The Netherlands). Isoflurane was used for inhalational anesthesia during measurements. The ear swelling was expressed as delta µm (ear thickness 1 h after whey challenge—basal ear thickness before i.d. challenge). To assess the severity of shock symptoms, 30 min after i.d. challenge a validated anaphylaxis symptom scoring table was used 23. The scoring was as follows: 0) no symptoms, 1) scratching nose and mouth, 2) swelling around eyes and mouth; pilo erection; reduced activity; higher breathing rate, 3) shortness of breath; activity after stimulation, 4) no activity after stimulation; shivering; muscle contractions, and 5) death by shock.

### Serum immunoglobulins, mMCP‐1, and galectin‐9

OVA‐specific IgE concentrations and galectin‐9 were measured in serum by means of ELISA as described previously 20, 24, 25. Results were analyzed with Microplate Manager PC software (Bio‐Rad Laboratories, Veenendaal, the Netherlands). Concentrations of OVA‐specific IgE in test sera were calculated in arbitrary units (A.U.) for each individual isotype, relative to a standard curve of pooled plasma. The concentration of mouse mast cell protease‐1 (mMCP‐1) in serum was determined 18 h after challenge by ELISA (Moredun Scientific Ltd., Penicuik, UK) according to the manufacturer's protocol. A reviewer commented that 18 h is a late timing for measuring an acute allergic response. Authors emphasize that mMCP‐1 levels remain high over a longer period and, therefore, are a true reflection of mucosal mast cell degranulation.

### T‐cell subsets and cytokine release

Single cell splenocyte and mesenteric lymph node (MLN) suspensions were obtained by passing the organs through a 70 µm filter. After red blood cell lysis, cells were blocked for 20 min in PBS containing 1% BSA and 5% FCS. 8 × 10^5^ cells were plated/well and incubated for 30 min at 4°C with different antibodies (eBioscience, Breda, the Netherlands or BD, Alphen aan den Rijn, the Netherlands, unless otherwise stated) against CD4, CD69, CXCR3, T1ST2, CD25, and isotype controls were used. Cells were fixed using 0.5% paraformaldehyde and permeabilized for intracellular staining with anti‐FoxP3 using the Foxp3 staining buffer set (eBioscience) according to the manufacturer's protocol. Flow cytometry was performed using FACS Canto II (BD, Alphen aan den Rijn, the Netherlands) and analyzed using FACSDiva software (BD). For cytokine release, splenocytes (8 × 10^5^/well) were incubated in RPMI1640 supplemented with penicillin (100 U/mL), streptomycin (100 µg/mL), and 10% FBS. Cells were stimulated for 5 days with 1 µg/mL anti‐CD3 or OVA (50 µg/mL). Supernatants were harvested for cytokine measurements by ELISA (IL10, IFNg, and IL5) according to the manufacturer's recommendations (eBioscience).

### Short chain fatty acids

The ceacal SCFA levels of acetic, propionic, butyric, isobutyric, and valeric acids were quantitatively determined as described previously 26, 27. The SCFA were captured using a Shimadzu GC2010 gas chromatograph (Shimadzu Corporation, Kyoto, Japan) equipped with a flame ionisation detector. SCFA concentrations were determined using 2‐ethylbutyric acid as an internal standard.

### Statistics

Data are presented as individual values or means ± SEM and were analyzed using one‐way ANOVA and post hoc Dunnet test using GraphPad Prism software (version 5.0). For anaphylaxis symptom scores, Kruskall–Wallis followed by Dunn's multiple comparison test was used.

## Results

### Acute allergic skin response and anaphylactic shock symptoms are reduced in sensitized mice fed the combination of scFOSlcFOS + *B. breve* M‐16V

**Figure 2:**
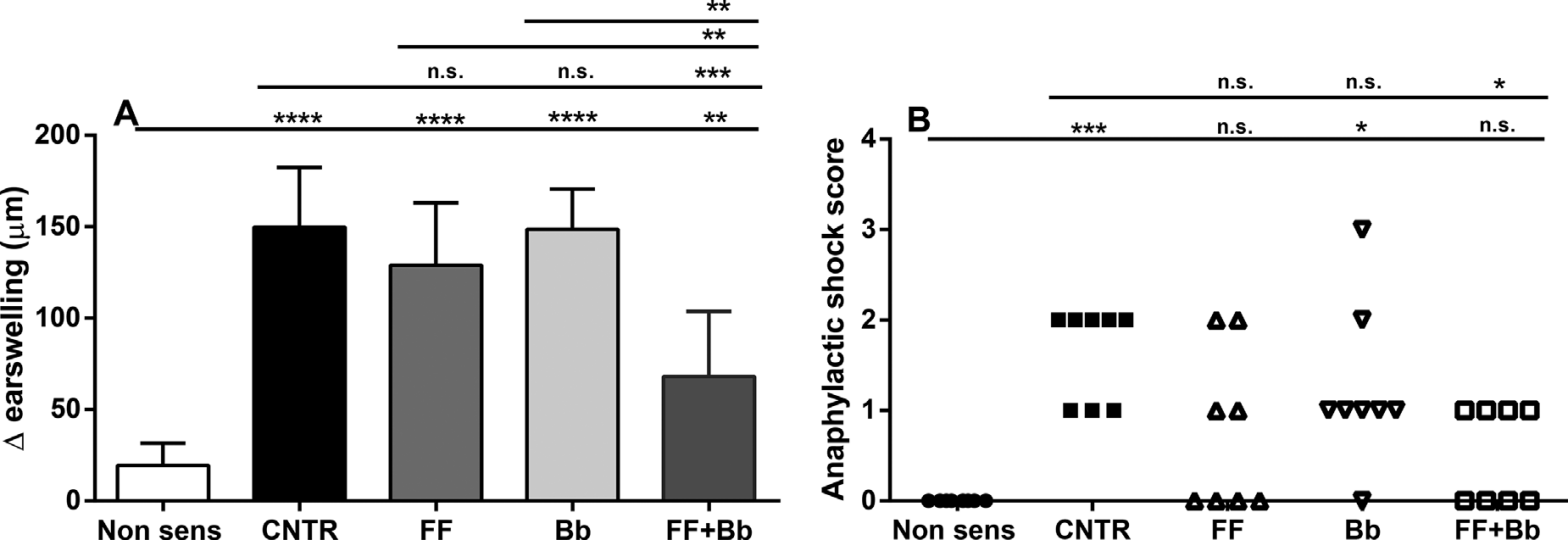
The effect of scFOSlcFOS (FF), *B. breve* (Bb), and the synbiotic diet containing scFOSlcFOS + *B. breve* (FF + Bb) on the (A) acute allergic skin response assessed upon intradermal challenge (delta ear swelling in µm) and (B) anaphylactic shock symptoms upon intradermal challenge (CNTR; OVA sensitized mice on control diet). Data are presented as Mean ± SEM (A) or individual values (B), *n* = 8. *p < 0.05, **p < 0.01, ***p < 0.001, and ****p < 0.0001. Data are analyzed using one‐way ANOVA followed by Dunnets multiple comparison test. Anaphylactic shock score: Kruskall–Wallis followed by Dunn's multiple comparison test.

To predict the possible beneficial effect of a dietary intervention with scFOSlcFOS + *B. breve* in already sensitized children, the non‐digestible oligosaccharides, pro‐ or synbiotics were fed to already sensitized mice (Fig. 1: Treatment setting). In mice fed a control diet, sensitization with OVA resulted in an acute allergic skin response upon i.d. challenge compared to non‐sensitized mice fed the control diet (CNTR: 149.8 + 11.5 vs. non‐sens: 19.3 + 4.3; Fig. 2A). Feeding the mice the diet supplemented with scFOSlcFOS (FF: 128.9 + 12.1) or *B. breve* (*Bb*: 148.6 + 8.3) from day 28, showed no effect on the acute allergic skin response compared to sensitized mice fed the control diet (CNTR). Interestingly, the scFOSlcFOS + *B. breve* combination significantly reduced the acute allergic skin response (FF + Bb: 68.2 + 12.6; *p* < 0.01) compared to mice fed either the scFOSlcFOS (FF: 128.9 + 12.1) or *B. breve* (Bb: 148.6 + 8.3).

In OVA sensitized mice, moderate to severe anaphylactic shock symptoms were observed 30 min. after intradermal OVA challenge, while the mice fed the scFOSlcFOS + *B. breve* supplemented diet showed just minor anaphylactic shock symptoms. No significant effects on shock symptoms were observed in mice fed the separate ingredients scFOSlcFOS or *B. breve* (Fig. 2B). Food intake and body weight measured weekly during the experiment did not differ between groups (data not shown).

### The synbiotic diet containing scFOSlcFOS + *B. breve* M‐16V reduced mast cell degranulation without significantly reducing allergen specific IgE levels

**Figure 3:**
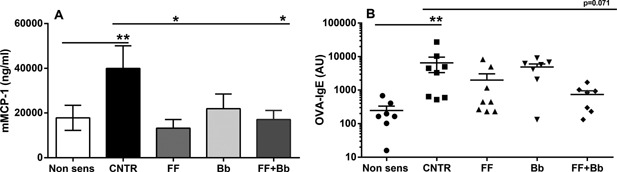
The effect of scFOSlcFOS (FF), *B. breve* (Bb), and the synbiotic diet containing scFOSlcFOS + *B. breve* (FF + Bb) on (A) mMCP‐1 concentrations and (B) OVA‐specific IgE in serum 18 hours after oral challenge (CNTR; OVA sensitized mice on control diet). Data are presented as individual values ± SEM (A) or Mean ± SEM (B), *n* = 8. *p < 0.05, **p < 0.01. Data are analyzed using one‐way ANOVA followed by Dunnets multiple comparison test.

To determine whether the scFOSlcFOS + *B. breve* supplemented diets were effective in reducing local mucosal mast cell degranulation if fed to already sensitized mice, mMCP‐1 serum concentrations were measured after oral OVA challenge. Serum mMCP‐1 concentrations (pg/ml) were increased in sensitized mice fed the control diet (CNTR: 40.0 + 10.1 vs. non‐sens: 17.8 + 5.6; *p* < 0.01; Fig. 3A). In mice fed the scFOSlcFOS (FF: 13.2 + 3.9; *p* < 0.05) or the scFOSlcFOS + *B. breve* (FF + Bb: 17.1 + 4.1; *p* < 0.05) supplemented diets, a significant reduction in mast cell degranulation was observed compared to the control diet (CNTR). In contrast, no significant effect was observed in mice fed the *B. breve* (Bb: 21.9 + 6.6; Fig. 3A). Although a functional effect was observed on mast cell degranulation, no significant effect but only a tendency toward reduced OVA‐IgE was observed in mice fed the scFOSlcFOS + *B. breve* (FF + Bb: 740.1 + 213.2 vs. CNTR: 6523 + 3175; *p* = 0.071; Fig. 3B).

### The combination of scFOSlcFOS + *B. breve* M‐16V changed cytokine profile in spleen cells

**Figure 4:**
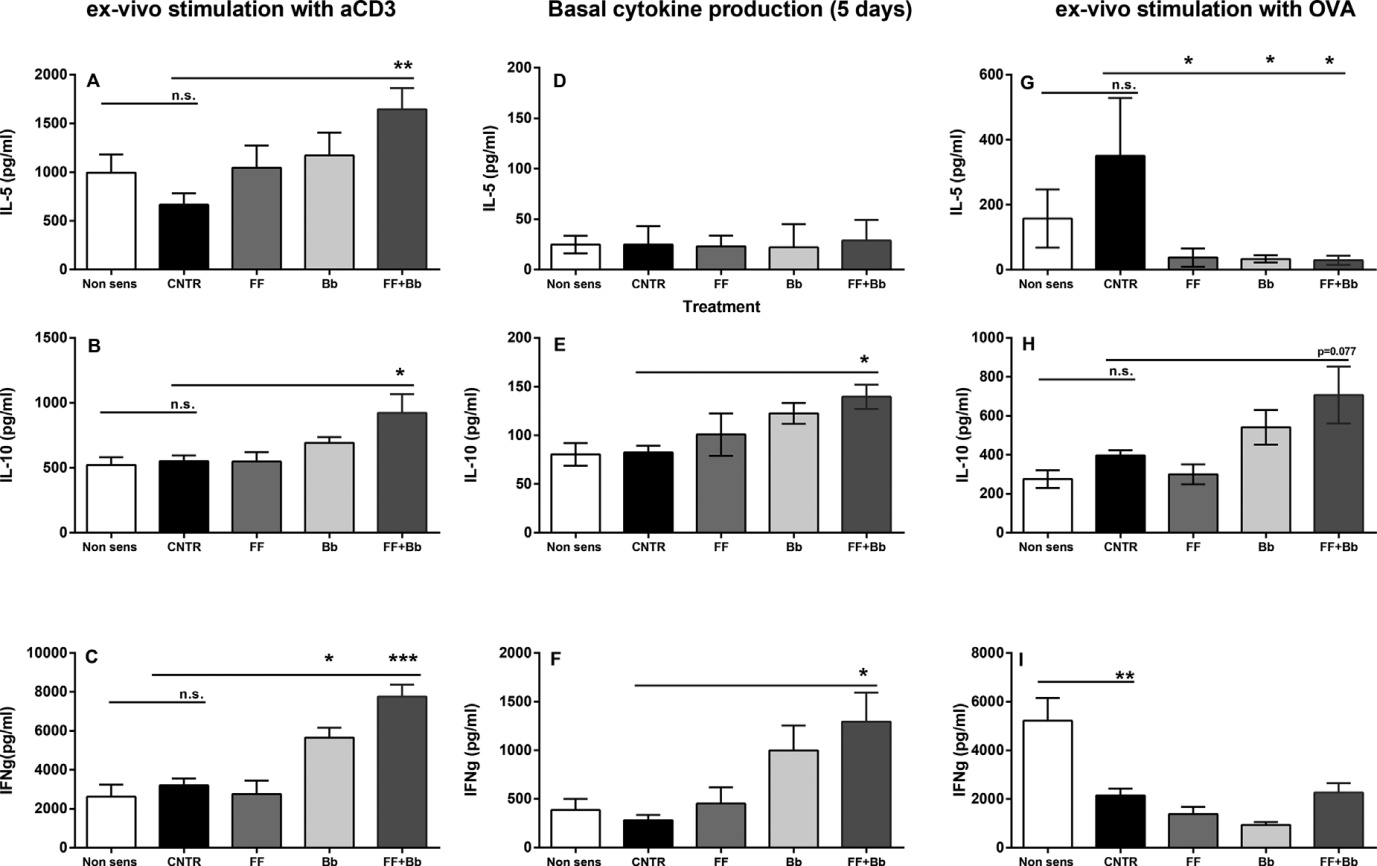
Cytokine release after ex vivo stimulation of splenocytes of mice fed the scFOSlcFOS (FF), *B. breve* (Bb), and the synbiotic diet containing scFOSlcFOS + *B. breve* (FF + Bb; CNTR; OVA sensitized mice on control diet). Eighteen hours after oral challenge IL5, IL10, and IFNg was determined in splenocytes stimulated ex vivo for 5 days with aCD3, medium, or OVA. Data are presented as Mean ± SEM, *n* = 8. *p < 0.05, **p < 0.01, ***p < 0.001. Data are analyzed using one‐way ANOVA followed by Dunnets multiple comparison test.

To study the mechanism underlying the effects of the scFOSlcFOS + *B. breve* supplemented diet in reducing allergic symptoms, cytokine production by T‐cells was studied. Polyclonal stimulation of T cells with aCD3 showed an increase in IL5, IL10, and IFNg in mice fed the scFOSlcFOS* + B. breve*, suggesting an increase in the number and/or activity of T cells in the spleen (Fig. 4A–C). Under basal conditions (no stimulation) splenocytes from mice treated with the scFOSlcFOS + *B. breve* diet showed an increase in the regulatory cytokine IL10 and the Th1 related cytokine IFNg. Under these conditions no IL5 production was observed (Fig. 4D–F). However, OVA stimulated splenocytes of supplemented mice showed a reduction in IL5 production in mice fed the scFOSlcFOS (FF: 37.6 + 28.4; *p* < 0.05), *B. breve* (Bb: 33.4 + 11.3; *p* < 0.05) or scFOSlcFOS + *B. breve* diet (FF + Bb: 29.1 + 14.3; *p* < 0.05) if compared to sensitized mice fed the control diet (CNTR: 350.9 + 178.0; Fig. 4G–I).

### Increased number of activated Th1‐cells in spleen after oral challenge in mice fed the combination of scFOSlcFOS + *B. breve* M‐16V

**Figure 5:**
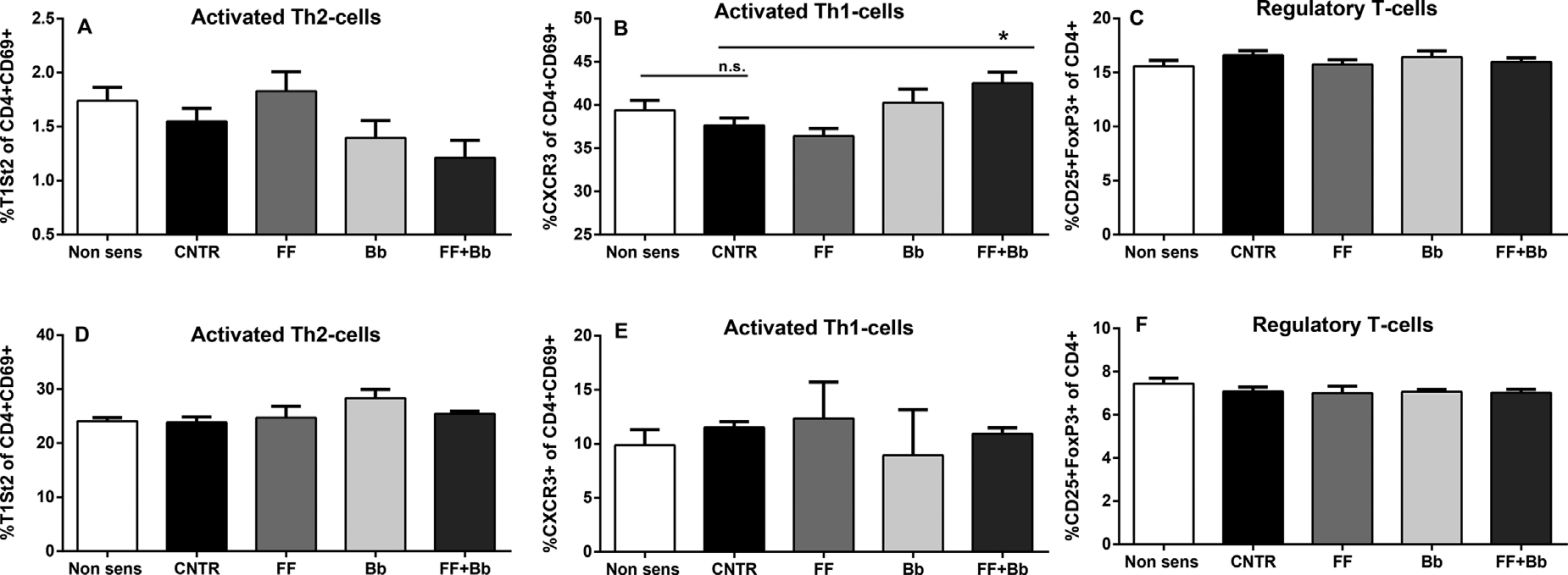
T‐cell subsets determined after oral allergen challenge of mice fed the scFOSlcFOS (FF), *B. breve* (Bb), and the synbiotic diet containing scFOSlcFOS + *B. breve* (FF + Bb; CNTR; OVA sensitized mice on control diet). Percentage of (A) activated Th2 and (B) activated Th1 cells were measured in splenocytes and MLN (D–E). Furthermore the percentage of FoxP3+ regulatory T cells was determined in spleen (C) and MLN (F). Data are presented as mean ± SEM, *n* = 6. *p < 0.05. Data are analyzed using one‐way ANOVA followed by Dunnets multiple comparison test.

To further determine the local effects of the dietary interventions, T‐cell subsets in the spleen and MLN were studied. Percentages of activated Th2‐, Th1‐, or regulatory T‐cells were not affected in sensitized mice compared to non‐sensitized mice (non‐sens) on a control diet (Fig. 5A–F). However, in sensitized mice fed the scFOSlcFOS + *B. breve* an increase in activated Th1‐cell percentages was observed in spleen (FF + Bb: 42.5 + 1.3 vs. CNTR: 37.6 + 0.8; *p* < 0.05; Fig. 5B) with no effects on the number of activated Th2‐cells or regulatory T cells (Fig. 5A and C). No effects were found on the percentages of activated Th2‐, Th1‐, or regulatory T‐cells in MLN (Fig. 5D–F) suggesting that there was an influence of the symbiotic mixture on T‐cells in systemic lymphatic tissue (spleen), but not in local lymphatic tissue (MLN).

### Increased production of short chain fatty acids (SCFA) in mice fed the combination of scFOSlcFOS + *B. breve* M‐16V

**Figure 6:**
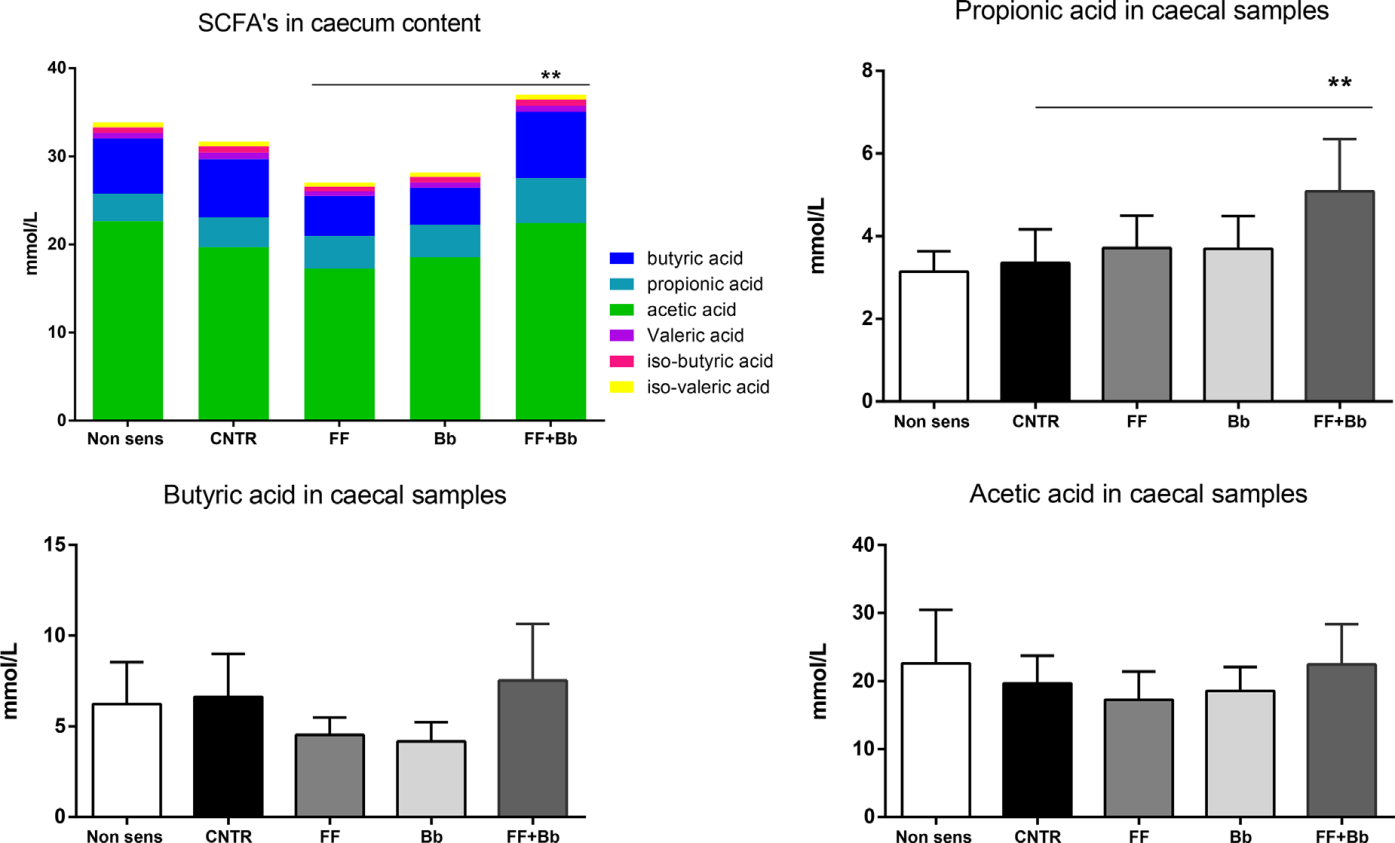
Total amount and diversity of short chain fatty acids (SCFA) were measured in ceacal samples of mice fed the scFOSlcFOS (FF), *B. breve* (Bb), and the synbiotic diet containing scFOSlcFOS + *B. breve* (FF + Bb; CNTR; OVA sensitized mice on control diet). Data are presented as means ± SEM, *n* = 6. *p < 0.01.

In order to connect the reduction in allergic symptoms by scFOSlcFOS + *B. breve* to microbial activity, we investigated the ceacal SCFA levels as bacterial fermentation products. In sensitized mice fed the symbiotic diet (FF + Bb), a significant increase in total SCFA levels were measured which can mainly be contributed to an increase in the SCFA proprionate (Fig. 6).

### Galectin‐9 concentrations in serum

No differences were observed on galectin‐9 serum concentrations in sensitized mice fed the dietary intervention post sensitization compared to sensitized mice fed the control diet (Supplement Fig. S2).

### scFOSlcFOS + *B. breve* M‐16V reduced the allergic response with increased serum galectin‐9 if an administration of the diets starts before sensitization (preventive setting)

In previous studies, it was shown that allergic symptoms (acute skin response, anaphylaxis) were reduced in mice fed a synbiotic mixture containing a lactose‐derived prebiotic mixture (scGOSlcFOS + *B. breve*) during oral sensitization and challenge, starting 14 days before the first sensitization 18, without effects on allergen‐specific IgE levels. Epithelium‐derived galectin‐9 was hypothesised as one of the regulatory mechanisms underlying this effect of the synbiotic mixture containing scGOS 19, 20. In the current study, reduced acute allergic skin responses and anaphylactic shock symptoms without affecting allergen‐specific IgE levels and an increase in epithelium derived galectin‐9 was measured in mice fed the synbiotic mixture in a preventive setting (scFoslcFOS + *B. breve*; Supplement Fig. S1A–D).

## Discussion

The current data show that a dietary intervention with a specific synbiotic concept reduces the magnitude of a murine allergic effector response after sensitization has taken place. This effect is shown on parameters that simulate clinical responsiveness (acute skin response, anaphylactic shock score) and on immunological effector and signaling molecules analyzed in serum and *ex vivo*, showing a consistent effect across different parameters. Unlike a variety of immunotherapy approaches that are frequent topics of immunological research, the specific synbiotic combination of non‐digestible oligosaccharides and *B. breve* M‐16V that was used in this study does not contain the allergen in any (modified) form or dose, but demonstrated potency to reduce an established allergic response. Similar effects were observed previously in a preventive setting, when dietary intervention was initiated before allergic sensitization 20, 28, 29.

It is hypothesized that such interventions modulate the immunological milieu in the gastrointestinal system and/or systemically, leading to reduced severity of specifically induced allergic responses 30, 31, 32, 33, 34, 35. Splenocyte restimulation results (Fig. 4) were consistent with a systemic immunomodulatory effect, showing altered cytokine profiles under basal conditions and after polyclonal T‐cell stimulation with anti‐CD3. The increase in the Th1 cytokine IFNg, and the increase in the regulatory cytokine IL10 (showing a trend in the antigen‐specific restimulated condition) in spleen. These findings resemble the effects described by de Kivit et al. 19, 20 where a combined Th1/regulatory T‐cell effect was implicated. Flow cytometry analysis in this study showed evidence of a relative increase in activated Th1 cells, but not in regulatory T cells (Fig. 5), leaving room to speculate that the activity of the regulatory T‐cell compartment may be modulated more intense than the number of cells.

Galectin 9 was previously shown to be correlated with reduced allergic responses in a preventive setting. These findings were reproduced in the current study (Supplement Fig. S2), but interestingly no correlation to galectin 9 levels was observed in a setting of established allergy. Galectin 9 is described to have IgE antagonizing effects 36 and to inhibit mast cell degranulation 37, but the current findings cannot be explained by this mechanism. Although the trend in reduced IgE levels may provide a mechanistic explanation for the reduction in the amplitude of the allergic response and the reduced marker of mast cell degranulation mMCP‐1, the modulatory effects within the T‐cell compartment and the interaction with mast cells may offer an alternative explanation.

In the intestinal tract, mast cells are mainly located in the lamina propria and they contribute both to tolerance for food as well as to sustain the immune response against pathogens 38. In allergic individuals, ingestions of harmless food proteins leads to the stimulation of allergen‐specific T‐helper 2 cells and the production of cytokines which are responsible for the production of allergen specific IgE. IgE binds to the high‐affinity receptor for IgE (Fc3RI) on mast cells. Allergen crosslinking of cell‐surface‐bound IgE leads to mast cell degranulation resulting in allergic symptoms. In addition to this well‐known and intensively investigated mechanism, mast cells are also able to establish interactions with helper T lymphocytes for antigen presentation and bidirectional cell–cell cooperation 39. T‐cell subsets have been reported to influence mast cell activation and to dampen mast cell mediated allergic responses, either via cell–cell contact 40, 41 or via cytokines. The regulatory T‐cell related cytokine IL10 reduced the release of cytokines in rat peritoneal mast cells 42 and mast cell degranulation in *in vivo* models 43, 44. Recently, it has been shown that constitutive Foxp3+ regulatory T‐cells can control mast cell activation and IgE‐dependent anaphylaxis in mice 45. These findings support the finding that cross‐talk between mast cells and T‐cell subsets and thereby a change in cytokine profiles underlie the reduction in anaphylactic shock symptoms, acute skin response and mast cell degranulation.

Microbes can also influence inflammatory responses in food allergy 34, 46 and particular the secondary effector phase of the allergic response. Patients suffering from allergic dermatitis display a different microbiota of the skin compared to healthy controls and in a mouse study it was shown that mast cells mediate *Staphylococcus aureus* delta‐toxin induced atopic dermatitis by attenuating mast cell activation and degranulation 46, 47. These findings show that a direct interaction between microbiota and mast cells and T‐cells might support the observed reduction in mast cell degranulation and allergic symptoms in mice fed the non‐digestible oligosaccharides and *B. breve*.

The increased and change in bacterial carbohydrate fermentation, reflected by increased levels of total short‐chain fatty acids (SCFA) and specifically propionic acid in mice fed the synbiotic diet indicates that the synbiotic diet induces specific changes impacting the composition and activity of the murine microbiome. Among SCFAs, acetic acid is relatively more readily produced than proprionic acid and butyric acid by most enteric and acetogenic bacteria. Propionic acid can be produced by three pathways from various sugar molecules or by metabolite cross‐feeding between gut microbes. Mostly species of the bacterial phyla *Bacteroidetes* and some of the *Firmicutes* are known producers of propionic acid in the intestine 48, which suggests that besides the *Bacterium bifido breve* M‐16V other species like *Bacteroidetes* and some *Firmicutes* might underlie at least in part the protective effects of the synbiotic diet.

An amino acid based formula supplemented with the plant derived non‐digestible oligosaccharides and *B. breve* was shown to support normal growth and to be safe in cow's milk allergic children 22. In the current study, using a pre‐clinical model to study the capacity of the specific synbiotic concept to reduce allergic symptoms, parameters that simulate clinical responsiveness were most effectively reduced. In contrast to the separate ingredients, only the combination of scFOSlcFOS and *B. breve* significantly reduced the acute allergic skin response in mice, which is considered a reflection of the skin prick test in humans. A similar outcome on the acute allergic skin response was observed in previous work using a specific combination of short‐chain galacto‐oligosaccharides, long‐chain fructo‐oligosaccharides, and *B. breve*, showing a synergistic effect of the combination in a preventive food allergy mouse model 18. The current study provides a pre‐clinical proof of concept that a specific synbiotic concept, targeting to modulate the intestinal microbiota but free of (modified) allergens, has the ability to reduce the magnitude of an established allergic response in the context of a mouse model for food allergy.

If the current findings translate successfully to a human application, it could provide a novel approach for the dietary management of individuals with severe cow's milk allergy by combining the safety of strict allergen avoidance with an immunomodulatory concept to stimulate tolerance acquisition and/or the outgrowth of cow's milk allergy. Clinical studies are ongoing to provide evidence on this subject.

## Conflict of Interest

Lucien Harthoorn and Paul Vos are employed by Nutricia Research. Johan Garssen and Betty van Esch are partly employed by Nutricia Research.

## Supporting information

Additional supporting information may be found in the online version of this article at the publisher's web‐site.

**Figure S1**. The effect of the synbiotic diet containing scFOSlcFOS and *B. breve* (FF + Bb) if fed before and during sensitization and challenge period (Preventive setting) on the (A) acute allergic skin response assessed upon intradermal challenge (delta ear swelling in µm) and (B) anaphylactic shock symptoms upon intradermal challenge.

**Figure S2**. The effect of scFOSlcFOS (FF), *B. breve* (Bb), and the synbiotic diet containing scFOSlcFOS + *B. breve* (FF + Bb) on galectin‐9 concentrations in serum 18 h after oral challenge.
