# Supplementary figures and images for: Post‐sensitization administration of non‐digestible oligosaccharides and Bifidobacterium breve M‐16V reduces allergic symptoms in mice
Source: Immun Inflamm Dis. 2016 Mar 24;4(2):155–65. doi: 10.1002/iid3.101 (PMC4879462; doi:10.1002/iid3.101)

Supplement FIGURE 1

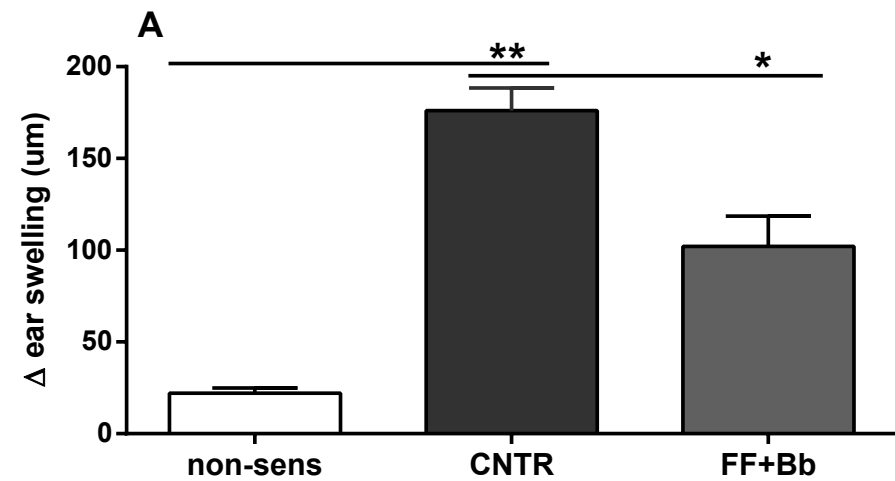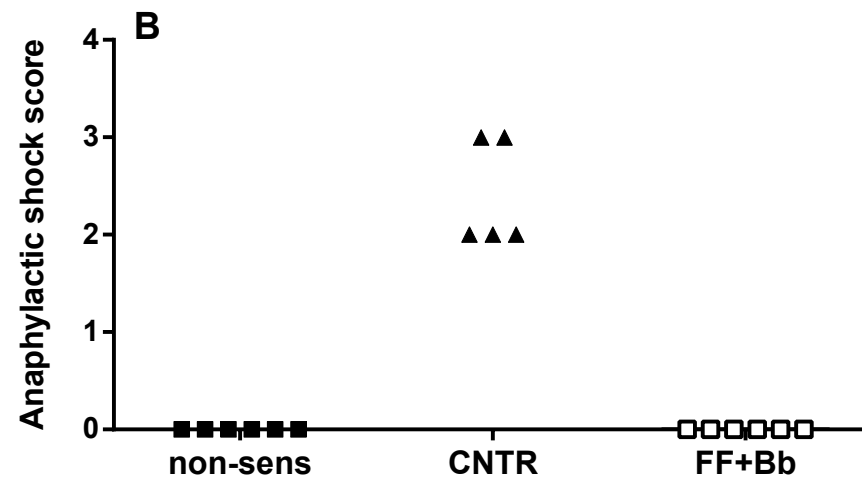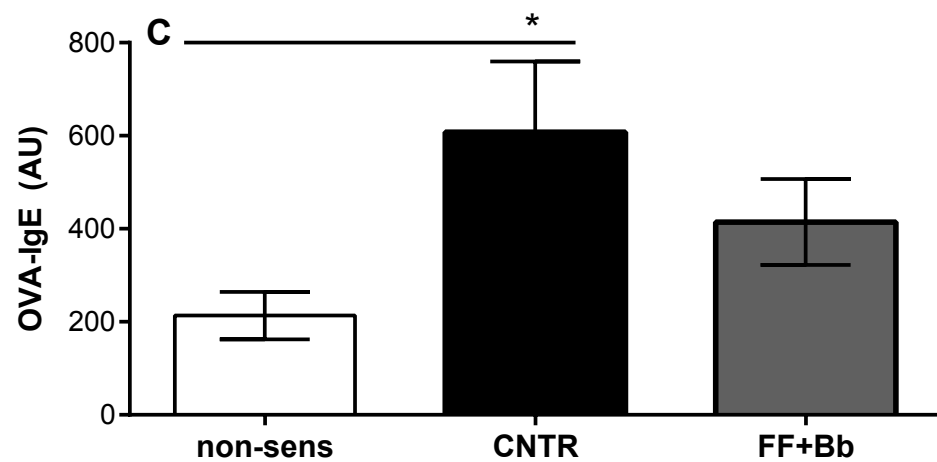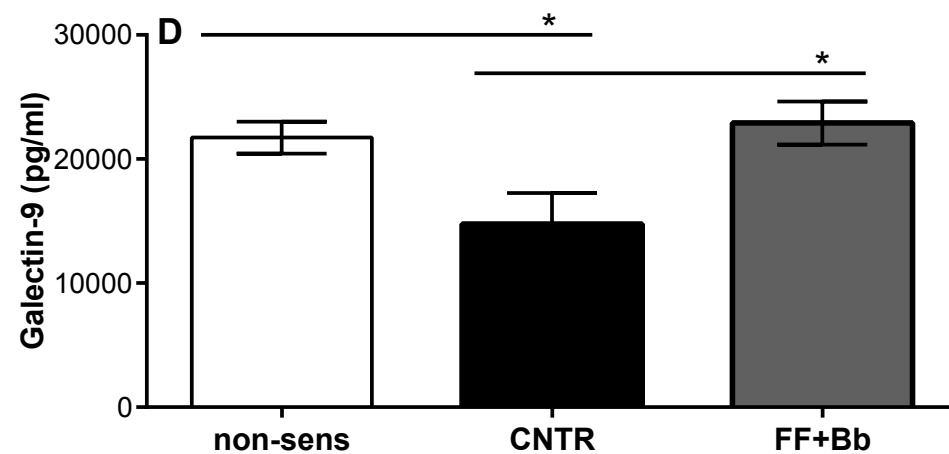

Supplement: Supplementary file 1 — Figure S1. The effect of the synbiotic diet containing scFOSlcFOS and B. breve (FF + Bb) if fed before and during sensitization and challenge period (Preventive setting) on the (A) acute allergic skin response assessed upon intradermal challenge (delta ear swelling in µm) and (B) anaphylactic shock symptoms upon intradermal challenge. [file IID3-4-155-s001.pdf]

## SUPPLEMENT FIGURE 2

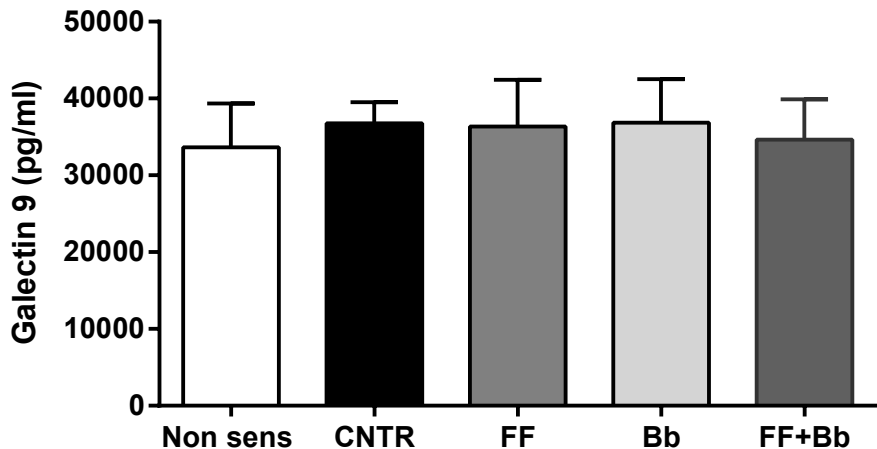

Supplement: Supplementary file 2 — Figure S2. The effect of scFOSlcFOS (FF), B. breve (Bb), and the synbiotic diet containing scFOSlcFOS + B. breve (FF + Bb) on galectin‐9 concentrations in serum 18 h after oral challenge. [file IID3-4-155-s002.pdf]
